# Hyperkalemia induced Brugada phenocopy

**DOI:** 10.1002/joa3.12498

**Published:** 2021-01-07

**Authors:** Janaki Rami Reddy Manne, Jalaj Garg

**Affiliations:** ^1^ Division of Cardiology Cardiac Arrhythmia Service Medical College of Wisconsin Milwaukee WI USA

**Keywords:** brugada phenocopy, hyperkalemia

## Abstract

We illustrate the case Brugada Type 1 pattern on electrocardiogram in a setting of hyperkalemia, changes which were reversible following normalization of serum potassium levels. Although Brugada Type 1 syndrome is associated with sudden cardiac death, a quick search for alternate reversible pathology is essential to timely management and avoid unnecessary cardiac intervention.

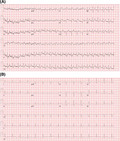

## 1 CASE PRESENTATION

**FIGURE 1:**
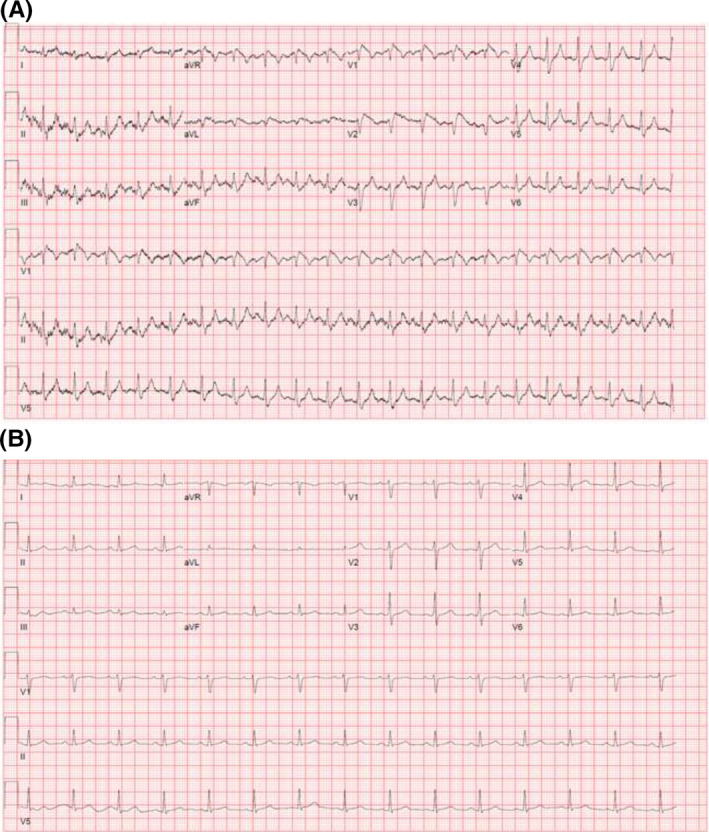
A, 12‐lead electrocardiogram demonstrating sinus tachycardia with ≥2 mm “coved type” ST‐segment elevations in leads V1‐V2 with T‐wave inversions (consistent with type 1 Brugada pattern). B, Repeat 12‐lead electrocardiogram 4 h later (following normalization of serum potassium level) demonstrating resolution of the Type 1 Brugada pattern

We present a case of a 34‐year‐old gentleman with no significant medical and family history presenting with severe left gluteal pain and lower extremity paresthesia several hours following a traumatic fall. On presentation his vitals were stable, and physical examination was unremarkable except for localized left gluteal region tenderness. A 12‐lead electrocardiogram (ECG) (Figure 1A) demonstrated ≥2 mm “coved type” ST‐segment elevations and T‐wave inversion in precordial leadsV1 and V2 (consistent with Type 1 Brugada pattern). Laboratory testing revealed potassium of 7.2 mEq/L (3.5–5 mEq/L), blood Urea Nitrogen 48 mg/dL (8–26 mg/dL), creatinine 3.2 mg/dL (0.5–1.5 mg/dL), sodium 148 mEq/L (135–145 mEq/L), chloride 108 mEq/L (98–108 mEq/L), serum bicarbonate 14 mEq/L (24–30 mEq/L), Creatinine kinase 161 350 units/L (<190 units/L) with a pH of 7.17.

## 2 DISCUSSION

Brugada Syndrome is an autosomal inherited channelopathy characterized by typical ST‐segment elevation in precordial leads V_1_‐V_3_ that predispose individuals to malignant ventricular arrhythmias and sudden cardiac death (SCD)^1^. Brugada Type 1 pattern is characterized by “coved‐type” ST‐segment elevation of at least 2 mm followed by a down‐sloping concave or rectilinear ST‐segment with a negative symmetric T‐wave. The Type 2 pattern is a “saddle‐shaped” ST‐segment elevation defined as a high take‐off (*r*′) that is ≥2 mm from the isoelectric baseline, followed by convex ST‐segment elevation with elevation ≥ 0.05 mV (with respect to the isoelectric baseline), with variable T‐wave in lead V1 and positive or flat T‐wave in lead V_2_.

Brugada Syndrome is characterized by Type 1 Brugada pattern (either spontaneous or induced) on ECG with at least one of the following high‐risk clinical features: documented ventricular arrhythmia (ventricular fibrillation, polymorphic ventricular tachycardia) or inducible ventricular arrhythmias on an electrophysiological study, family history of SCD (less than 45 years old), documented Type 1 Brugada ECG pattern in family members, syncope, or nocturnal agonal respiration.^2^ Abnormal repolarization as a result of ionic imbalance in the epicardium [reduced inward positively charged ionic currents (Na^+^ and Ca^2+^) and exaggerated outward currents (K^+^)] more pronounced in the right ventricular outflow tract (RVOT) generates a transmural voltage gradient thus manifesting as the characteristic Brugada ECG patterns.^3^ Although mutations in genes encoding various ion channels have been identified, most commonly implicated were the loss‐of‐function mutations in SCN5A (mutation identified in only 20%‐30% of BrS probands).^4^

A transient Brugada‐like ECG pattern in the absence of clinical features of Br is termed as Brugada phenocopy (BrP). BrP has been previously described in association with inferior/right ventricular myocardial infarction, pulmonary embolism, metabolic/electrolyte abnormalities (hypokalemia, metabolic acidosis, hyponatremia, and hyperkalemia), and adrenal insufficiency.^3^ Although hyperkalemia produces classical electrocardiographic manifestations, only small subsets of patients develop BrP. Thus, differentiating these two clinical entities is essential as implantable cardioverter‐defibrillator is not warranted in BrP. The diagnostic criteria^4^ suggestive of BrP are shown in Table 1. The clinical implications of BrP remain unknown, although a recent study demonstrated an increased risk of malignant arrhythmias with BrP.^5^ Consequently, BrP treatment includes the management of the underlying condition. Extracellular hyperkalemia, by decreasing the resting membrane potential and inactivation of the sodium channels, can reproduce BrP in susceptible individuals.^4^ Unlike congenital BrS, the ion channel dysfunction in hyperkalemia‐induced BrP is transient, a finding which is not reproducible with sodium channel blocking agents.

**TABLE 1:** Brugada phenocopy diagnostic criteria

The electrocardiogram (ECG) pattern has a type 1 or type 2 Brugada morphology The patient has an identifiable underlying condition The ECG pattern resolves after the resolution of the underlying condition There is a low clinical pretest probability of true Brugada syndrome determined by a lack of symptoms, medical history, and family history Negative provocative testing with sodium channel blockers such as ajmaline, flecainide, or procainamide Provocative testing not mandatory if surgical RVOT manipulation has occurred within the last 96 h The results of genetic testing are negative (desirable but not mandatory because the SCN5A (sodium channel voltage‐gated type V alpha‐subunit mutation) is identified in only 20%‐30% of probands affected by true Brugada Syndrome

The hyperkalemia was treated with intravenous calcium gluconate, insulin, glucose, and aggressive fluid resuscitation. He subsequently underwent emergent fasciotomy and debridement for the left gluteal muscle, left lower extremity anterior and lateral muscle compartment syndrome. The transthoracic echocardiogram showed normal left ventricular function with no regional wall motion abnormalities. Subsequently, normalization of serum potassium resulted in the resolution of ST‐segment elevations within 4 hours, as noted on the 12‐lead ECG (Figure 1B). As our patient had no prior history of palpitations, unexplained syncope, or family history of SCD and with spontaneous resolution of Brugada ECG pattern with hyperkalemia correction, no further provocative challenging or electrophysiological study was performed.

Our patient illustrates the case BrP in a setting of hyperkalemia, which was reversible following normalization of serum potassium levels. Since, hyperkalemia can unmask familial BrS, a thorough history taking (including a detailed family history and exclusion of BrS) is needed especially in young males like this case. Additionally, a quick search for alternate reversible pathology is essential for timely management and to avoid unnecessary cardiac intervention.

## CONFLICT OF INTEREST

Authors declare no conflict of interests for this article.
